# Vitamin B12 measurements across neurodegenerative disorders

**DOI:** 10.1186/s40734-020-00085-8

**Published:** 2020-03-12

**Authors:** Nijee S. Luthra, Ariane H. Marcus, Nancy K. Hills, Chadwick W. Christine

**Affiliations:** 1 Department of Neurology, University of California, San Francisco, 1635 Divisadero, Suite 520-530, San Francisco, CA 94115 USA; 2 Department of Neurology, University of California, San Francisco, 400 Parnassus Avenue, San Francisco, CA 94122 USA; 3 Department of Epidemiology and Biostatistics, University of California, San Francisco, 550 16th Street, San Francisco, CA 94158 USA

**Keywords:** Vitamin B12, Cyanocobalamin, Subacute combined degeneration

## Abstract

**Background:**

Vitamin B12 deficiency causes a number of neurological features including cognitive and psychiatric disturbances, gait instability, neuropathy, and autonomic dysfunction. Clinical recognition of B12 deficiency in neurodegenerative disorders is more challenging because it causes defects that overlap with expected disease progression. We sought to determine whether B12 levels at the time of diagnosis in patients with Parkinson’s disease (PD) differed from those in patients with other neurodegenerative disorders.

**Methods:**

We performed a cross-sectional analysis of B12 levels obtained around the time of diagnosis in patients with PD, Multiple System Atrophy (MSA), Dementia with Lewy Bodies (DLB), Alzheimer’s disease (AD), Progressive Supranuclear Palsy (PSP), Frontotemporal Dementia (FTD), or Mild Cognitive Impairment (MCI). We also evaluated the rate of B12 decline in PD, AD, and MCI.

**Results:**

In multivariable analysis adjusted for age, sex, and B12 supplementation, we found that B12 levels were significantly lower at time of diagnosis in patients with PD than in patients with PSP, FTD, and DLB. In PD, AD, and MCI, the rate of B12 decline ranged from − 17 to − 47 pg/ml/year, much greater than that reported for the elderly population.

**Conclusions:**

Further studies are needed to determine whether comorbid B12 deficiency affects progression of these disorders.

## Background

Vitamin B12 deficiency is common in aging and causes a number of neurological conditions including cognitive and psychiatric disturbances, gait instability, neuropathy, and autonomic dysfunction [1–5]. Because features of B12 deficiency overlap with those seen in neurodegenerative diseases, clinical recognition of this deficiency is difficult and requires blood measurement.

We recently measured B12 levels at baseline in early, untreated Parkinson’s disease (PD) patients from the DATATOP study [6] and found that 5% had deficient level (< 212 pg/ml) and 13% had borderline low level (< 250 pg/ml) [7]. We also found that those participants in the lowest B12 tertile had greater annualized worsening of gait and balance as assessed by the ambulatory capacity score compared to those in the middle or upper B12 tertiles [8]. B12 levels, but not homocysteine, methylmalonic acid, or holotranscobalamin, were associated with greater gait impairment. Because the DATATOP study was conducted in the late 1980’s, it is not known if these rates are accurate in contemporary patients.

Since in our prior study hematological indices (mean corpuscular volume and hematocrit) did not help identify patients with low B12 levels, knowledge of B12 levels among neurodegenerative conditions could increase the diagnostic rate of B12 insufficiency in patients with PD and thus allow for appropriate treatment. In this exploratory study, we retrospectively analyzed B12 measurements across various neurodegenerative diseases obtained around the time of diagnosis and evaluated the rate of B12 decline in patients with PD compared to patients with a variety of other neurodegenerative disorders.

## Methods

### Study design and protocol

The study was approved by Committee on Human Research at University of California, San Francisco (UCSF). We searched electronic medical records between 2007 and 2015 to identify all patients > 40 years old seen in the UCSF Department of Neurology with a recorded B12 measurement, obtained as part of routine clinical care, at the UCSF Clinical laboratory. During the timeframe of 2007–15, the UCSF laboratory used the same Vitamin B12 Chemiluminescent Assay (ADVIA Centaur System, Siemens). At the low end of the normal range 207 pg/ml, this assay has a 4% within–run coefficient of variation (CV) and a 4.4% run-to-run CV. In order to obtain an estimate of endogenous B12 levels and because levels > 1999 pg/ml are not quantified and only resulted as such, we excluded patients with levels greater than the UCSF laboratory upper limit of normal (> 911 pg/ml) because these high levels likely reflected excessive supplementation of B12. As clinical B12 deficiency may occur at or above the laboratory cutoff (211 pg/ml), we used a cut-off of < 250 pg/ml to identify patients at higher risk for developing B12 deficiency [7]. Only patients with a B12 measurement within ±3 years of neurodegenerative disease diagnosis were included in the analysis. Remaining records were reviewed to confirm the year of diagnosis and if and when levodopa was started. Because treatment with metformin and proton pump inhibitors are associated with reduced B12 levels while treatment with B12 and/or a multivitamin increases B12 levels, we reviewed the medical record to determine whether patients had these exposures documented at the time of the blood draw for B12.

We included the following diagnoses: PD, AD, MSA, DLB, PSP, FTD and MCI. Patients were categorized according to the diagnosis they received at their latest neurology visit. In order to develop cohorts with as typical a clinical phenotype as possible, those with a diagnosis of mixed dementia (i.e. vascular dementia plus AD) were excluded. To evaluate the rate of B12 decline (change in B12 levels/year), we identified patients diagnosed with PD, AD, or MCI and who had at least two B12 measurements > 6 months apart, with the first measurement within 3 years of diagnosis. Since B12 levels in the elderly have been shown to gradually decline over time [9] and in order to estimate the endogenous rate of decline of B12, we excluded patients with > 10% increase between first and second measurements since such an increase would likely reflect that the patient had begun B12 supplementation between measurements.

### Statistical analysis

Statistical analyses were performed using Stata version 15.1 (StataCorp, College Station, TX). Patient characteristics across diagnosis groups were compared using Fisher’s exact tests for categorical variables and analysis of variance (ANOVA) for continuous variables. We first examined covariates potentially associated with B12 levels using univariate linear regression models. Variables significant at the 0.05 level in univariate analysis were then included in a multivariable model. Although neither age nor sex was significantly associated with B12 levels in univariate analysis, we also included these in the multivariable model. In those with MCI, AD and PD who had more than one B12 measurement, differences between the two measurements were calculated and divided by time elapsed to estimate median rates of decline for each group. These rates were compared using a non-parametric Kruskal-Wallis test. A significance threshold of 0.05 was used.

## Results

### Vitamin B12 levels at baseline

Of those diagnosed with predominant neurocognitive symptoms (AD, MCI, FTD, DLB), 16% (220/1312) were excluded due to a B12 level > 911 pg/ml. Of those evaluated for predominant parkinsonism (PD, MSA, PSP), 12% (84/691) were excluded due to having a B12 level > 911 pg/ml. A total of 713 patients met criteria to be included in the analysis. Group characteristics across diagnostic categories were compared using ANOVA. Age and sex differed significantly across diagnostic groups (Table 1). B12 levels at time of diagnosis also varied significantly. Although patients with PD were more likely to have B12 levels< 250 pg/ml, this difference was not significant. Neither use of a multivitamin nor a separate B12 supplement was different across groups. As expected, levodopa use was more common among those diagnostic groups with parkinsonism (PD, DLB, MSA, and PSP). The overall percentage of patients on metformin, a medication associated with a decline in B12, was low but use was significantly different across groups, with the DLB group having the greatest metformin use. Use of proton pump inhibitor was not significantly different across groups. (Table 1).

**Table 1:** Characteristics of cohort

	AD (***n*** = 204)	DLB (***n*** = 36)	FTD (***n*** = 24)	MCI (***n*** = 290)	MSA (***n*** = 9)	PD (***n*** = 130)	PSP (n = 20)	***P***-value*
Age at diagnosis, mean (sd)	74.4 (9.7)	73.4 (8.6)	68.4 (8.6)	70.4 (9.9)	66.1 (3.6)	68 (10.2)	69.9 (6.3)	< 0.001**
Age at time of B12, mean (sd)	73.8 (9.7)	73 (8.6)	67.7 (8.5)	69.8 (9.9)	65.1 (3.8)	68.4 (9.7)	69 (6.1)	< 0.001**
Female (%)	118 (57.8)	17 (47.2)	11 (45.8)	150 (51.7)	3 (33.3)	51 (39.2)	9 (45)	0.04
**B12 level (pg/ml), mean (sd)**	**465.1 (165.5)**	**518.7 (180.4)**	**542.9 (159.3)**	**499.4 (180.7)**	**549.8 (145.6)**	**454.7 (168.4)**	**539.1 (178.1)**	0.015**
Patients with B12 < 250 pg/ml (%)	13 (6.4)	2 (5.6)	0	18 (6.2)	0	13 (10.0)	1 (5.0)	0.69
Medications (%)								
Levodopa	1 (0.5)	20 (55.6)	0	0	5 (55.6)	116 (89.2)	9 (45)	< 0.001
Multivitamin	58 (28.4)	6 (16.7)	6 (25)	85 (29.3)	1 (11.1)	23 (17.7)	3 (15.0)	0.10
Vitamin B12	16 (7.8)	3 (8.3)	2 (8.3)	18 (6.2)	0	2 (1.5)	2 (10.0)	0.13
Metformin	11 (5.4)	7 (19.4)	1 (4.2)	27 (9.3)	0	4 (3.1)	2 (10.0)	0.02
PPII	28 (13.7)	8 (22.2)	4 (16.7)	54 (18.6)	0	16 (12.3)	5 (25.0)	0.27

Abbreviations: *MCI* mild cognitive impairment; *PD* Parkinson’s disease; *MSA* multiple systems atrophy; *DLB* dementia with Lewy bodies; *AD* Alzheimer’s disease; *PSP* progressive supranuclear palsy; *FTD* frontotemporal dementia; *PPI* proton pump inhibitor

^*^calculated using Fisher’s exact test unless otherwise indicated

^**^calculated using analysis of variance

In univariate analyses examining the potential association of covariates with B12 levels around the time of disease diagnosis, levels of B12 in MCI, FTD, MCI and PSP were all found to be significantly higher on average than levels in those with PD (Table 2). No differences were observed between B12 levels in patients with PD and those with either AD or MSA. Neither age nor sex were associated with B12 levels, although the 95% CI for age indicated a higher likelihood of decreasing levels with increasing age. Both multivitamin use and B12 supplementation were significantly associated with increased B12 levels. Neither use of metformin or a proton pump inhibitor was associated with significantly lower B12 levels.

**Table 2:** Univariate linear regression of factors associated with B12 levels at time of diagnosis

Diagnosis	Coefficient	(95% CI)	P-value
Parkinson’s disease	Ref		
Alzheimer’s disease	10.4	(−27.7, 48.5)	0.59
Dementia with Lewy bodies	63.9	(−0.06, 128)	0.05
Frontotemporal dementia	88.2	(12.7, 164)	0.02
Mild cognitive impairment	44.6	(9.77, 80.5)	0.015
Multiple systems atrophy	95.0	(−22.1, 212)	0.11
Progressive supranuclear palsy	84.4	(2.74, 166)	0.04
Female gender	−9.56	(−35.2, 16.1)	0.47
Age at diagnosis	−0.99	(−2.28, 0.29)	0.13
Multivitamin use	91.1	(62.4, 120)	< 0001
B12 supplementation	88.7	(35.3, 142)	0.001
Metformin	23.0	(−26.3, 72.3)	0.36
Proton Pump Inhibitor	0.62	(−34.2, 35.5)	0.97

In multivariable analysis adjusted for sex, age, multivitamin use and B12 supplementation, patients with DLB, FTD and PSP all had higher B12 levels on average than those with PD, although MCI was no longer significant (*p* = 0.068; Table 3). Multivitamin use and B12 supplementation were independently associated with increased B12 levels.

**Table 3:** Multivariable analysis of factors associated with B12 level at time of diagnosis

Diagnosis	Coefficient^*^	(95% CI)	P-value
Parkinson’s disease (PD)	Ref		
Alzheimer’s disease (AD)	1.90	(−36.4, 40.2)	0.92
Dementia with Lewy bodies (DLB)	64.3	(1.94, 127)	0.04
Frontotemporal dementia (FTD)	76.4	(3.24, 149)	0.04
Mild cognitive impairment (MCI)	32.8	(−2.39, 68.0)	0.068
Multiple systems atrophy (MSA)	100.4	(−12.8, 214)	82
Progressive supranuclear palsy (PSP)	81.6	(2.55, 161)	0.04
Female gender	−7.65	(−32.6, 17.3)	0.55
Age at diagnosis	−0.86	(−2.14, 0.41)	0.185
Multivitamin use	93.5	(65.0, 122)	< 0.001
B12 supplementation	86.4	(34.3, 139)	0.001

*In a linear regression, coefficients are interpreted as follows: a one unit increase in x results in an increase in B12 pg/ml of (coefficient) pgs. For example, the coefficient associated with DLB is interpreted: Compared to patients with PD, patients with DLB have on average a B12 level that is 64.3 pg/ml higher at the time of diagnosis

### Rate of vitamin B12 decline

**Fig. 1:**
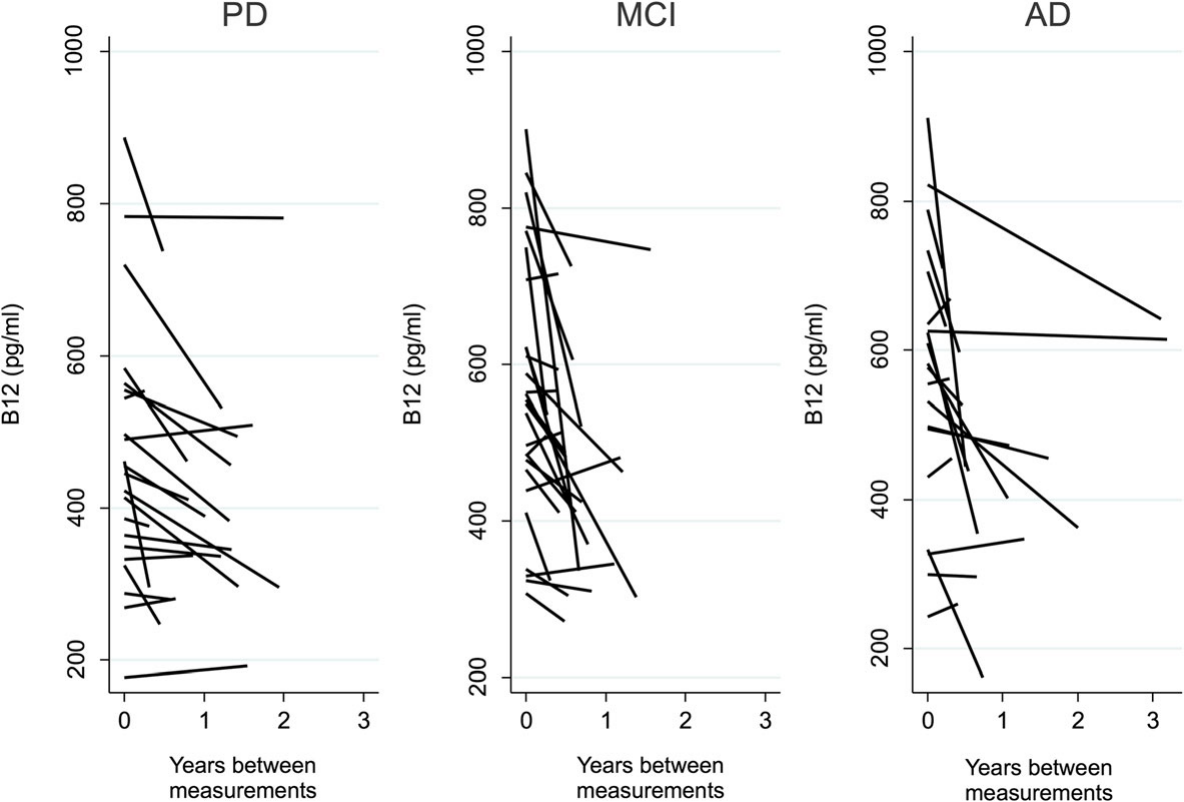
Rate of B12 Decline in PD, MCI, and AD patients. Plots showing individual declines of B12 in patients with PD, MCI, and AD. Median (interquartile range) rate of decline were − 17 (−35, − 0.4), −47 (−112, −8), and − 29 (− 124, 2.4) pg/ml/year for PD, MCI, and AD, respectively

Of those diagnosed with predominant neurocognitive symptoms (AD, MCI, FTD, DLB), 16% were excluded due to the first B12 > 911 pg/ml and 23% due > 10% increase in the second B12 measurement compared to baseline. Of those evaluated for predominant parkinsonism, 14% were excluded due to the first B12 > 911 pg/ml and 27% due to an increase of > 10% at the second measurement. Since there are fewer patients with two B12 measurements, we confined the analysis of the change in B12 level/year across the PD, MCI and AD groups, and a total of 69 patients were included in the analysis (Fig. 1). Median (interquartile range) rate of decline for those with PD, MCI, and AD were − 17 (− 35, − 0.4), − 47 (− 112, − 8), and − 29 (− 124, 2.4) pg/ml/year, respectively. Rate of decline did not differ significantly across groups.

## Discussion

In this cohort of patients with neurodegenerative conditions, we found that B12 levels obtained within 3 years of neurological diagnosis were significantly lower in patients with PD than in patients with PSP, FTD, and DLB when adjusted for sex, age, and B12-supplementing medications. The rate of B12 decline in PD, MCI, and AD ranged from 17 to 47 pg/ml/year, far in excess of the approximately 5 pg/ml/year measured in a study of normal elderly adults [9]. These findings are of clinical importance since we have previously found an association of low B12 levels with more rapid development of gait impairment in PD [7] but is also relevant in patients with other neurodegenerative diseases, who are expected to develop cognitive impairment and/or gait instability due to disease progression. Failure to identify and treat this deficiency at diagnosis or in the years after diagnosis may well increase patient morbidity.

As expected, B12 supplementation and multivitamin use were associated with higher B12 levels. Multivitamin use ranged from 11 to 29% while separate B12 supplement use ranged from 1.5–10%. These rates were similar to the rates of use of about 41 and 5%, respectively, in a recent study of a large PD cohort [10] and were not statistically different across groups. Both the age of diagnosis and percentage of females were lower in PD and MSA, but linear regression models showed that age and sex were not associated with B12 levels.

One of the limitations of this study is that we did not have information on patient body mass index (BMI) and nutrition risk index. Obesity and elevated BMI have been shown to be associated with lower vitamin B12 levels [11], whereas in hospitalized patients at nutritional risk, high B12 levels (> 1000 pg/nl) have been shown to be associated with increased mortality [12]. In contrast, our study targeted outpatients with early neurodegenerative disease excluded patients with levels over 911 pg/ml, and is therefore was different patient population. However, since patients with advanced neurodegenerative diseases are at higher risk for impaired nutritional status, future studies of moderately advanced neurodegenerative disease should evaluate associations of B12 with mortality.

A systematic literature review testing for an association of low vitamin B12 levels with development of cognitive impairment has been inconclusive [13], although studies using more specific biomarkers of low B12 status (methylmalonic acid and holotranscobalamin) have shown significant associations [14]. While in our study, the B12 levels were lower and comparable in PD and AD patients, Boston et al. (2019) have shown that vitamin B12 levels were not significantly lower in AD patients compared to healthy controls [15]. One reason for the apparent difference in our findings may be that we only looked at patients within 3 years of AD diagnosis. Our study also lacks a healthy control group for comparison.

In PD particularly, lower B12 levels might be more likely to develop for a number of reasons, including change in dietary intake of B12 (some adopt a low-protein diet), *Helicobacter pylori* infection, delayed gastric emptying and development of constipation, which is associated with bacterial overgrowth [16]. Finally, the prevalence of borderline low B12 (< 250 pg/ml) in 10% of PD patients in this cohort from 2007 to 2015 was similar to what was observed in the DATATOP study (13%) [6], suggesting that it remains a fairly common comorbidity in PD.

Our prior study showing that low B12 levels predicted greater progression of gait instability did not demonstrate similar predictive characteristics associated with other B12 analytes traditionally used to establish a diagnosis of B12 deficiency, including methylmalonic acid, homocysteine and holotranscobalamin [7]. This observation raises the question as to whether B12 levels might have a more direct effect on PD progression. Interestingly, Schaffner and colleagues recently reported that B12 directly modulates leucine–rich repeat kinase 2 (LRRK2) [17]. Mutations in LRRK2 account for about 5% of hereditary PD [18] and the most common variant (G2019S) has been established to increase LRRK2 activity [19, 20]. Since Schaffner and colleagues found that higher levels of B12 inhibit LRRK2 kinase activity, their work suggests that low B12 levels may directly contribute to PD pathogenesis by allowing for increased LRRK2 activity [21].

While aggregation of specific proteins (α-synuclein and β-amyloid) are defining characteristics PD and AD pathology, the role of these proteins in the early pathogenesis of the sporadic forms of these disorders is not clear and has led some to hypothesize that insight into disease modifying targets requires discovery of other biomarkers present at disease onset and subsequent subgroup analysis according to these candidate biomarkers [22, 23]. Data from this study, showing that B12 levels are lower in PD than other neurodegenerative diseases, along with recent clinical [7] and biochemical data [17] suggest that B12 warrants further study as an early PD biomarker.

Of note in hospitalized patients, high vitamin B12 levels have been associated with increased in-hospital mortality [12, 24–26]. This may be because cobalamin is an acute phase reactant, and elevated cobalamin levels have been reported in patients with oncologic diseases, hepatic disease, and in the elderly, hospitalized and critically ill medical patients who were not taking B12 supplements [12, 27]. Since it is unknown whether there is a relationship between high vitamin B12 levels and mortality in patients with neurodegenerative disorders, future studies should explore this question.

To our knowledge, this is the largest study to assess B12 levels across early neurodegenerative diseases. However, our study is limited by its observational design and small sample size for the less common disorders. Because the study was retrospective and was based in the Movement Disorder and the Behavioral Neurology clinics at UCSF, a suitable control group with B12 measurements was not available. Although B12 measurements are commonly obtained in the initial evaluation of patients with gait or cognitive conditions, in this study B12 measurements were drawn as part of usual clinical practice. It is therefore possible that initial or repeat B12 levels were more likely to be measured in those who developed neuropathy, possibly causing ascertainment bias and/or an overestimate of annualized decline in B12 levels. Also, since supplement use was based on retrospective chart review, this information is likely to be less accurate than that collected as part of a prospective study. Finally, although we were not able to correlate disease stage with B12 levels, because the analysis was restricted to patients with B12 levels measured within 3 years of diagnosis, these patients were predominantly in early disease stages.

## Conclusion

Our finding of a lower B12 level around the time of diagnosis in PD compared to PSP, FTD, and DLB and greater rates of B12 decline in those with PD, AD and MCI supports the need for increased vigilance for B12 deficiency in these conditions. Future studies are needed to confirm these findings and determine whether low B12 is associated with more rapid disease progression and if so, whether early B12 supplementation reduces morbidity.

## Data Availability

The data that support the findings of this study are available from the authors upon reasonable request.

## Abbreviations

AD: Alzheimer’s disease
DLB: Dementia with Lewy Bodies
FTD: Frontotemporal Dementia
LRRK2: Leucine–rich repeat kinase 2
MCI: Mild Cognitive Impairment
MSA: Multiple System Atrophy
PD: Parkinson’s disease
PSP: Progressive Supranuclear Palsy
UCSF: University of California, San Francisco
